# Developmentally controlled changes during Arabidopsis leaf development indicate causes for loss of stress tolerance with age

**DOI:** 10.1093/jxb/eraa347

**Published:** 2020-08-28

**Authors:** Aakansha Kanojia, Saurabh Gupta, Maria Benina, Alisdair R Fernie, Bernd Mueller-Roeber, Tsanko Gechev, Paul P Dijkwel

**Affiliations:** 1 School of Fundamental Sciences, Massey University, Palmerston North, New Zealand; 2 Center of Plant Systems Biology and Biotechnology, Plovdiv, Bulgaria; 3 Department Molecular Biology, Institute of Biochemistry and Biology, University of Potsdam, Potsdam, Germany; 4 Max Planck Institute of Molecular Plant Physiology, Potsdam-Golm, Germany; 5 Department of Plant Physiology and Molecular Biology, University of Plovdiv, Plovdiv, Bulgaria; 6 University of Birmingham, UK

**Keywords:** Abiotic stress, ageing, age-related changes, *Arabidopsis*, development, oxidative stress, senescence, stress-responsive metabolites

## Abstract

Leaf senescence is the final stage of leaf development and is induced by the gradual occurrence of age-related changes (ARCs). The process of leaf senescence has been well described, but the cellular events leading to this process are still poorly understood. By analysis of progressively ageing, but not yet senescing, *Arabidopsis thaliana* rosette leaves, we aimed to better understand processes occurring prior to the onset of senescence. Using gene expression analysis, we found that as leaves mature, genes responding to oxidative stress and genes involved in stress hormone biosynthesis and signalling were up-regulated. A decrease in primary metabolites that provide protection against oxidative stress was a possible explanation for the increased stress signature. The gene expression and metabolomics changes occurred concomitantly to a decrease in drought, salinity, and dark stress tolerance of individual leaves. Importantly, stress-related genes showed elevated expression in the early ageing mutant *old5* and decreased expression in the delayed ageing mutant *ore9*. We propose that the decreased stress tolerance with age results from the occurrence of senescence-inducing ARCs that is integrated into the leaf developmental programme, and that this ensures a timely and certain death.

## Teaser

Intrinsic stress levels increase as part of the normal leaf developmental programme and this helps the plant to decide which leaves to sacrifice for whole-plant survival during environmental stress.

## Introduction

Plant ageing is an irreversible and continuous process whereby plants ultimately enter their last phase in life, known as senescence (Jing *et al.*, 2003; Lim *et al.*, 2007). Leaf senescence is the final stage of leaf development, during which nutrients are recycled from the deteriorating leaf to other growing parts of the plant (Hörtensteiner, 2006; Guiboileau *et al.*, 2010). A change of leaf colour from green to yellow due to chlorophyll degradation is the first visible indication of senescence (Mattila *et al.*, 2018). Thus, ageing is an essential part of the normal plant developmental programme, whereby sequential changes occur during the plants’ life, from germination to senescence and finally death. The progressive changes that occur during plant development are termed age-related changes (ARCs) (Jing *et al.*, 2005; Jibran *et al.*, 2013; Kanojia and Dijkwel, 2018). Examples of visible ARCs in plants are the transition of juvenile to adult leaves, the initiation of the reproductive phase, and the yellowing of senescing leaves. Endogenous ARCs include cell division, cell expansion, changes in hormone levels, degradation of cellular components, and many more. Leaf development is achieved through ARCs and, concomitant with its emergence, expansion, and maturation, the increase in leaf age leads to the progressive decline of cell and tissue function and eventually leaf senescence (Caswell and Salguero-Gomez, 2013; Kanojia and Dijkwel, 2018) It is possible that some ARCs play specific roles in ageing and the induction of senescence and here we term such changes as senescence-inducing ARCs.

While several studies have focused on transcriptomic and metabolomic changes in fully developed and senescent leaves (Buchanan-Wollaston *et al.*, 2003; Breeze *et al.*, 2011; Woo *et al.*, 2018), ageing is a continuous process and senescence-inducing ARCs may already take place in young, still expanding leaves. Therefore, this research attempts to examine the occurrence of early senescence-inducing ARCs and their effects on maturing Arabidopsis leaves of different ages. Using Arabidopsis wild type (WT) and ageing mutants, we found that a reduction in intrinsic stress tolerance is part of the normal leaf development programme. We propose that this allows individual leaves of a plant to respond to environmental stress in an age-dependent manner, ultimately benefitting whole-plant survival.

## Materials and methods

### Plant growth conditions

Arabidopsis WT accessions Landsberg *erecta* (L*er*-0) and Columbia (Col-0), the early senescence mutant *old5* (*onset of leaf death5*), and the delayed senescence mutant *ore9* (*oresara 9*, kindly provided by the Hye Ryun Woo group, Pohang University of Science and Technology, South Korea) were used in this study. Plants were grown in a growth chamber under controlled conditions [21 °C, 65% relative humidity, 16 h/8 h (light/dark) photoperiod, 180 µE light intensity]. Plant age was counted in days after germination (DAG) whereby 1 DAG defines the first day on which green cotyledons became visible.

### Sample harvest and RNA preparation

The Arabidopsis L*er*-0 first rosette leaf pair from 10 (early expanding), 15 (mid expanding), and 20 DAG (fully expanded) were harvested for RNA sequencing. Three biological replicates were used for each time point. Samples were snap-frozen in liquid nitrogen, RNA was extracted using the Ambion PureLink RNA-mini Kit, and DNA was removed using the Ambion TURBO DNA-free Kit (Thermofisher Scientific), according to the manufacturer’s instructions. Approximately 1 µg of each RNA sample was sequenced using Illumina technology (HiSeq 4000, 100 bp paired-end; BGI Tech Solutions, Hong Kong).

### RNA sequencing analysis

Raw sequencing reads exhibited a very high quality (assessed using FastQC http://www.bioinformatics.babraham.ac.uk/projects/fastqc; Supplementary Table S1 at *JXB* online). Reads were filtered to remove rRNA contamination using SortMeRNA v2.1 (Kopylova *et al.*, 2012) and pseudoaligned to the Arabidopsis transcriptome (TAIR10), using kallisto v0.42.4 (Bray *et al.*, 2016) with 100 bootstraps. R-package Sleuth v0.28.1 (Pimentel *et al.*, 2017) was used for differential expression analysis. A gene was considered significantly differentially expressed if the b-value was ≥1 and the adjusted *P*-value was ≤0.1. Clustering of significantly differentially expressed genes (DEGs) was performed using the pheatmap package in R/Bioconductor (Kolde, 2015). Transcripts per million values were used for plotting heat maps for selected sets of genes.

Gene Ontology (GO) enrichment was performed by submitting the AGI codes of selected genes to the ThaleMine database (https://bar.utoronto.ca/thalemine/begin.do;Krishnakumar *et al.*, 2015). Significantly enriched GO terms were selected using a false discovery rate (FDR) cut-off of 0.05. Enrichment analysis of DEGs was performed with previously published stress-, senescence-, and hormone-related genes (Kreps, 2002; Harb *et al.*, 2010; Breeze *et al.*, 2011; Rasmussen *et al.*, 2013), using the Overlap package in R/Bioconductor which uses Fisher’s exact test (Shen and Sinai, 2019).

### Transcript analysis using quantitative real-time PCR (qRT-PCR)

The rosette leaves of the first leaf pair were detached at 10, 15, and 20 DAG and snap-frozen in liquid nitrogen. Samples were ground to a fine powder and total RNA was isolated using the Zymo Research (California, USA) Plant RNA Miniprep Kit and treated with DNase I (Roche Applied Sciences, Switzerland) according to the manufacturer’s instructions. RNA samples were converted to cDNA utilizing oligo(dT) primers and using the Transcription First Strand cDNA Synthesis Kit (Roche). SYBR Green PCR master mix (Roche) was used for qRT-PCRs using LightCycler 480 multiwell plates and a LightCycler 480 II (Roche). Reaction conditions were: 95 °C for 5 min, 35 cycles of 95 °C for 10 s, 60 °C for 10 s, 72 °C for 10 s, and a final step of 72 °C for 4 min. Primer efficiency measurements and quantification cycles (Cq) were calculated using LinRegPCR software (v.2015.0, http://LinRegPCR.nl). *ACTIN 2* (*ACT2*), *TUBULIN BETA-2* (*TUB2*), and *UBIQUITIN-PROTEIN LIGASE 7* (*UPL7*) genes were used as reference genes. QuantPrime (Arvidsson *et al.*, 2008) was used for primer design (Supplementary Table S2). Relative mRNA expression was determined using the Pfaffl method (Pfaffl, 2004).

### Metabolite profiling

First rosette leaf pairs of L*er*-0 plants were detached 10, 15, and 20 DAG, ground to a fine powder, and stored at –80 °C. A 50 mg aliquot of ground tissue of each sample was analysed by gas chromatography-mass spectrometry (GC-MS). Five biological replicates were used for each sample. Metabolite profiling was conducted using the method described in Lisec *et al.* (2006). Given that the extraction buffer used in this method does not conserve the redox state of ascorbate, the measured dehydroascorbate generally reflects the total ascorbate pool. Chromatograms and mass spectra were evaluated using ChromaTOF4.5 (Leco), Tag Finder 4,2 software (Luedemann *et al.*, 2008), and Xcalibur 2,1 (Thermo Fisher Scientific, Waltham, MA, USA). The MPI Golm Metabolome Database (http://gmd.mpimp-golm.mpg.de) was used as a comparative resource for peak quantification and annotation (Kopka *et al.*, 2005).

### Stress treatments

For the drought treatment experiments, field capacity of the pots was measured before planting, using the saturation method described by Samarah (2005). Germinated plants were well watered every alternate day, but water was withheld after 8, 13, and 18 DAG. After 6 d of drought stress, the samples were used for analyses (Supplementary Fig. S1A). To assess the water status of drought-stressed plants, relative water content (RWC) was measured by the method of Yamasaki and Dillenburg (1999). For the salinity shock experiments, plants were watered normally until 8, 13, and 18 DAG. Then at 10, 15, and 20 DAG, plants were watered with 300 mM NaCl every alternate day (Supplementary Fig. S2). After 6 d, samples were used for analyses. Chlorophyll content was determined using *N*,*N*'-dimethylformamide as described (Wellburn, 1994). For dark stress experiments, plants at 10, 15, and 20 DAG were transferred to a dark box at 21 °C for 4 d and subsequently moved back to the growth chamber for a 3 d recovery period.

## Results

### Global transcriptomic changes in immature Arabidopsis leaves

**Fig. 1.**
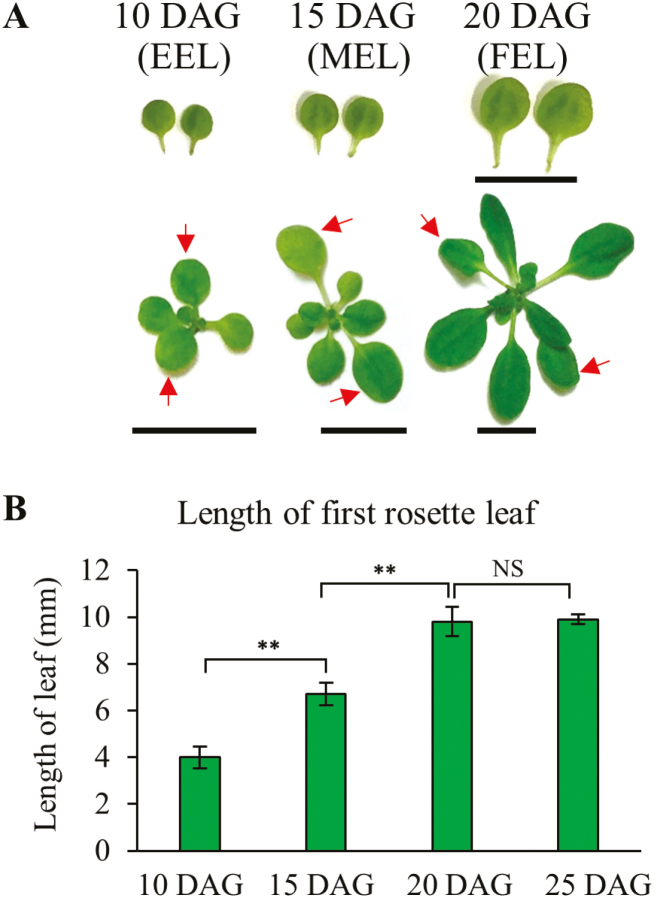
First rosette leaf pair expansion. (A) Arabidopsis WT first rosette leaf pairs detached from plants at 10, 15, and 20 days after germination (DAG). Scale bar=10 mm. (B) The length of first rosette EELs (early expanding leaves), MELs (mid expanding leaves), and FELs (fully expanded leaves) from plants grown under a long-day photoperiod were measured at 10, 15, 20, and 25 DAG. ** indicates a significant difference between the indicated values at *P*≤0.01 determined by ANOVA; NS, not significant.

To obtain a global picture of expression-linked ARCs in expanding Arabidopsis leaves, comparative transcriptome analysis using RNA sequencing was performed. Arabidopsis WT L*er*-0 plants were grown in a long-day photoperiod and the first leaf pair of the rosette was harvested at 10 DAG (early expanding leaves, EELs). Mid expanding leaves (MELs) were harvested 15 DAG and fully expanded leaves (FELs) at 20 DAG. These different ages were selected based on the developmental stage of the first rosette leaf, where EELs had attained a length of 4 mm and MELs about twice that length at 7 mm. At 20 DAG, FELs reached their final size at ~10 mm longitudinally (Fig. 1A, B). At the time of the final harvest, the reproductive phase (Koornneef *et al.*, 1994) and leaf senescence had not yet initiated (Supplementary Fig. S3).

**Fig. 2.**
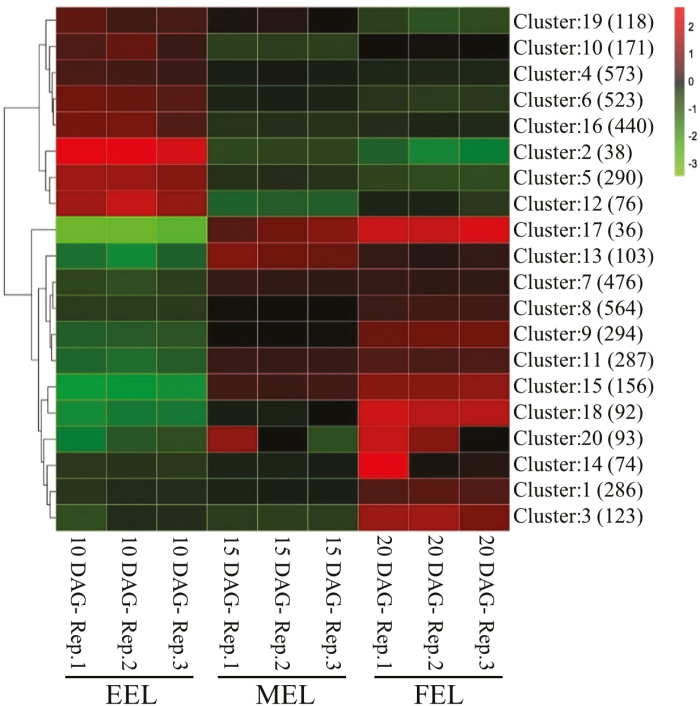
Heat map of differentially expressed genes. Heat map depicting gene expression patterns of Arabidopsis WT 10, 15, and 20 DAG first rosette leaves. Three biological replicates were used for each time point. Gene expression clusters are indicated to the right with number of genes in each cluster in parentheses. Green represents decreased and red increased gene expression. The colour scale represents mean-centred log_2_-normalized transcripts per million values. Rep., replicate.

RNA sequencing reads were generated, and DEGs were identified from WT EELs, MELs, and FELs as described in the Materials and methods. Figure 2 shows a heat map representing clusters of 4813 significant DEGs between EEL, MEL, and FEL samples. Out of all DEGs, 2229 were down-regulated and 2584 up-regulated (Supplementary Table S3). Eight clusters in EEL were up-regulated and seven down-regulated as compared with MELs and FELs. Five clusters contained genes which were up-regulated only in FELs. The robustness of the transcriptomic data was tested by analysing the expression of individual genes by qRT-PCR in independent biological replicates (Supplementary Fig. S4). The patterns of expression changes of the selected genes were similar to those identified by RNA sequencing. Thus, these results present the patterns of ARCs taking place in first rosette EEL, MEL, and FEL samples.

### Gene Ontology enrichment of differentially expressed genes

In the above section, the RNA sequencing data revealed transcriptional changes taking place in Arabidopsis first rosette leaves well before the onset of senescence. Next, DEG profiles enriched in specific biological functions were identified to discover GO terms that change during early leaf development.

**Fig. 3.**
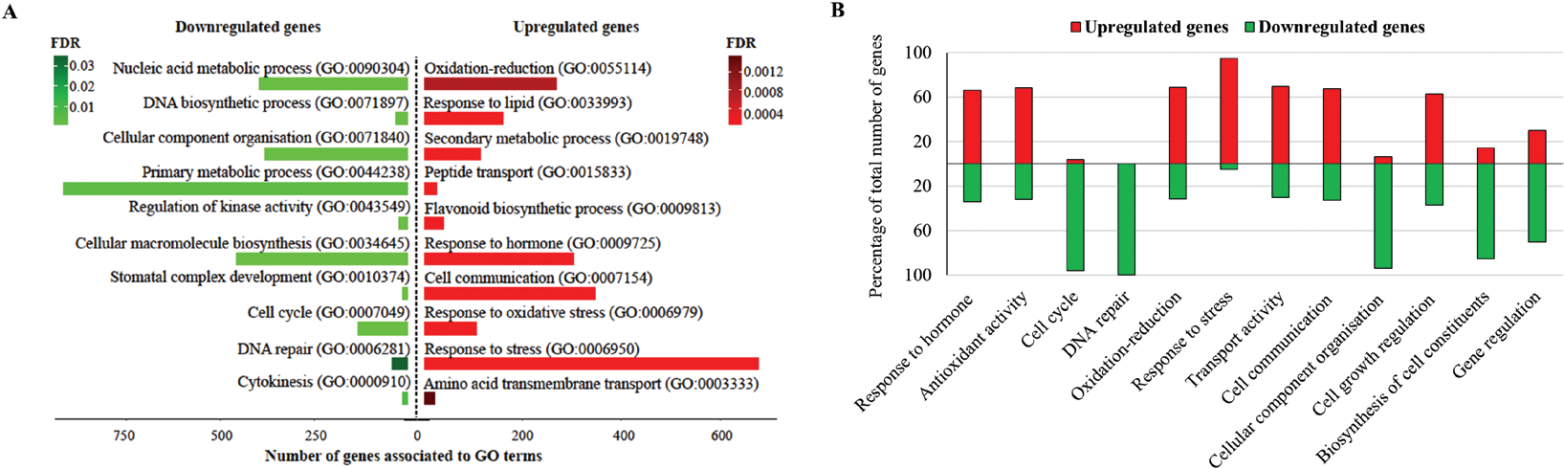
Differentially expressed down- and up-regulated genes. (A) GO enrichment of differentially expressed genes. The numbers of down- and up-regulated genes associated with a GO term are shown on the *x*-axis. Bars are coloured based on the FDR values. Only selected GO terms in the category of biological processes are shown in this graph (a detailed list of GO enrichment is given in Supplementary Table S4). (B) Percentage of total up- and down-regulated genes enriched in specific biological functions in MEL and FEL, as compared with EEL samples. Green and red bars represent down- and up-regulated genes, respectively.

GO enrichment of the DEGs was performed (Krishnakumar *et al.*, 2015) and enrichment of up-regulated and down-regulated genes in various biological processes is shown in Fig. 3A and Supplementary Table S4. Based on GO enrichment, the percentage of up-regulated and down-regulated genes in MEL and FEL samples of specific biological functional groups was calculated (Fig. 3B). Because harvested leaves were still actively growing, we expected GO enrichment of genes involved in growth, development, and general metabolism. However, we also found striking GO enrichment of genes that were not obviously linked to growth, but rather to senescence. Of note, stress-related (Breeze *et al.*, 2011; Kanojia and Dijkwel, 2018) and hormonal changes (Jibran *et al.*, 2013; Khan *et al.*, 2014) have been linked to plant ageing, and genes and GO terms involved in these processes were conspicuously up-regulated (Fig. 3B).

Thus, results from the GO enrichment analysis pinpoint crucial genes in stress and hormone biology that gradually occur as Arabidopsis leaves grow older. Therefore, these genes were selected for more detailed analysis.

### Up-regulation of stress-responsive genes with age in developing leaves

The highest percentage of up-regulated genes in MEL and FEL samples were those related to stress responses (Fig. 3B), and this indicates that cellular stress is changing in MEL and FEL samples compared with EELs. The list of genes responsive to stress was studied in more detail to identify the categories of stress-related genes.

**Fig. 4.**
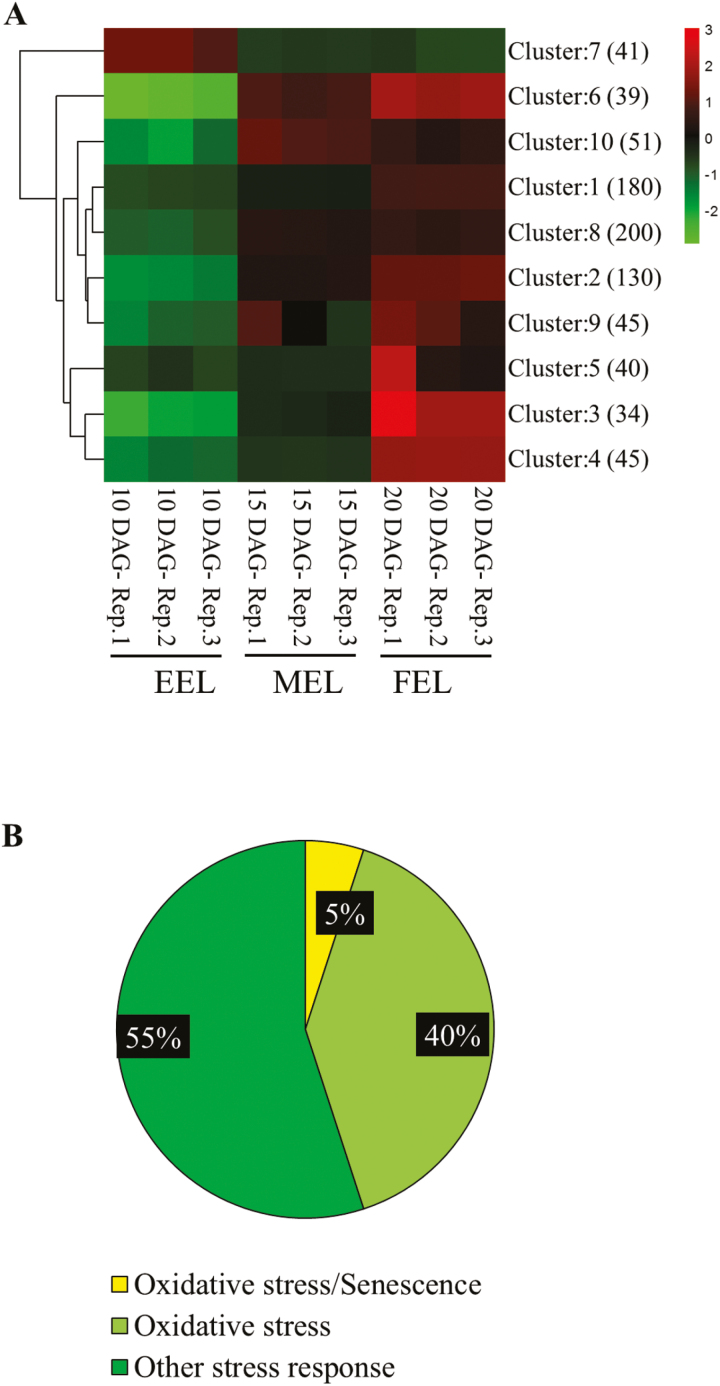
Differentially expressed genes involved in stress responses. (A) Heat map depicting differentially expressed stress-responsive genes in 10, 15, and 20 first rosette leaf samples. Green and red colour represent down- and up-regulated genes, respectively. Gene expression clusters are indicated to the right with the number of genes in each cluster in parentheses. The colour scale represents mean-centred log_2_-normalized transcripts per million values. Rep., replicate. (B) Pie chart representing the percentage of up-regulated oxidative stress/senescence-, oxidative stress-, and other stress-responsive genes.

The heat map in Fig. 4A depicts the pattern of stress-related gene expression in EELs, MELs, and FELs. The results show that among the 805 stress-related DEGs, just one cluster of 41 genes was down-regulated, while all other clusters, encompassing 764 genes, were up-regulated in MEL and FEL samples. Interestingly, in cluster 7, most of the genes (25 out of 41) are involved in defence responses. Moreover, examination of the biological function of up-regulated genes showed that 38 (5%) are linked to oxidative stress and leaf senescence, 324 (40%) to oxidative stress, and the remaining genes (55%) are related to different stress conditions (Fig. 4B; Supplementary Table S5). The up-regulated genes modulating leaf senescence and oxidative stress include a large number of NAC and WRKY transcription factors that are known to play a prominent role in the response to stress during the onset of senescence (Balazadeh *et al.*, 2008). The up-regulated senescence-associated genes include *SAG2*, *SAG13*, *SAG14*, *SAG15*, *SAG20*, *SAG21*, *SAG29*, and *SAG113*. Genes belonging to mitogen-activated signalling pathways (*MPK3*, *MPK11*, *MKK1*, and *MAPKKK18*) and members of tetraspanin (*TET3*, *TET8*, and *TET9*) were also up-regulated. Taken together, the results suggest that cellular stress or the capacity to cope with stress changes during leaf development.

### Up-regulation of genes involved in stress-related hormone pathways

**Fig. 5.**
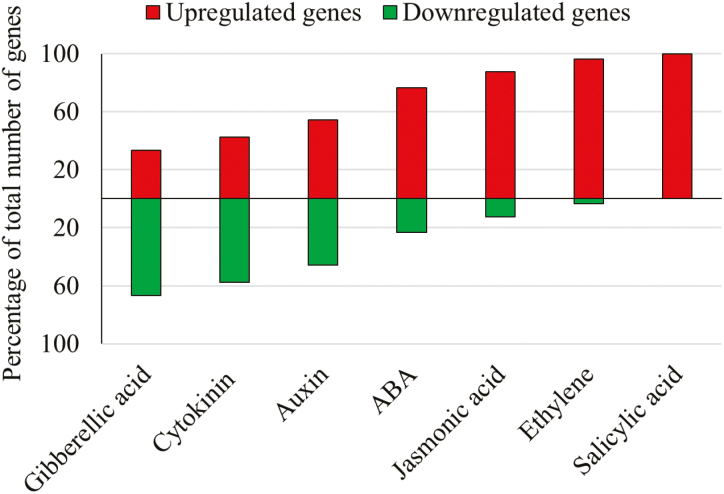
Differentially expressed genes involved in hormone biosynthesis and signalling. Bar graph representing the percentage of down- and up-regulated differentially expressed genes in MEL and FEL as compared with EEL samples. Green and red bars represent down- and up-regulated genes, respectively.

Hormones play various roles in plant developmental processes and the regulation of stress and senescence. Comparing the number of DEGs involved in the biosynthesis and signalling of each hormone may indicate the type of cellular changes occurring throughout leaf development. In total, 185 DEGs were linked with biosynthesis and signalling of different hormones. The graph in Fig. 5 presents the percentage of up-regulated and down-regulated genes in MEL and FEL samples associated with specific hormones. DEGs associated with gibberellic acid and cytokinin were mostly down-regulated, while DEGs associated with auxin were up-regulated and down-regulated in similar numbers. Crucially, gibberellic acid and cytokinin were shown to function in supressing senescence (Greenboim-Wainberg, 2005). In contrast, DEGs linked to the hormones abscisic acid (ABA), jasmonic acid (JA), ethylene, and salicylic acid (SA) were mostly up-regulated.

Out of 30 genes involved in ABA biosynthesis and signalling, 23 genes were over-represented in MEL and FEL samples (Fig. 5). The key ABA biosynthesis genes, *NCED3* (*NINE-CIS-EPOXYCAROTENOID DIOXYGENASE3*), *ZEP* (*ZEAXANTHIN EPOXIDASE*), and *AAO3* (*ABSCISIC ALDEHYDE OXIDASE3*) (Endo *et al.*, 2014), were up-regulated in MEL and FEL samples, suggesting an increased level of ABA in these tissues. Expression of *CYP707A4* (cytochrome P450, family 707), which plays a predominant role in ABA catabolism, especially under stress conditions (Kushiro *et al.*, 2004), was also up-regulated. Several other genes responsive to ABA, specifically during stress, were found to be up-regulated with leaf age (Supplementary Table S3).

JA is another hormone known to have an active role in plant growth, response to stress, and during leaf senescence (Santino *et al.*, 2013). The EEL samples showed low expression of genes regulating JA biosynthesis and signalling, while 21 out of 24 genes in this group displayed up-regulation in MEL and FEL samples. These up-regulated genes comprise *AOS* (*ALLENE OXIDE SYNTHASE*), *AOC1*, *AOC2*, *AOC3* (*ALLENE OXIDE CYCLASE*), *LOX1*, *LOX2*, *LOX3*, and *LOX4* (*LIPOXYGENASE*), all of which encode enzymes that catalyse important steps in the biosynthesis of JA (Stenzel *et al.*, 2012). The other over-represented genes that regulate chief roles in JA signalling are *JAZ1*, *JAZ3*, *JAZ5*, *JAZ6*, *JAZ7*, *JAZ9*, *JAZ10* (*JASMONATE-ZIM DOMAIN*), *MYC2* [encoding a basic helix–loop–helix (bHLH) transcription factor], and *COI1* (*CORONATINE INSENSITIVE1*).

Transcriptomic results showed a total of five DEGs, involved in signalling and biosynthesis of SA, up-regulated in MELs and FELs compared with EELs. These highly expressed SA-related genes included *ICS1* (*ISOCHORISMATE SYNTHASE1*), *PBS3* (*AVRPPHB SUSCEPTIBLE3*), *EDS1*, *EDS5* (*ENHANCED DISEASE SUSCEPTIBILITY*), and *PAD4* (*PHYTOALEXIN DEFICIENT4*).

Ethylene is a well-known senescence-inducing hormone in plants (Schaller, 2012; Jibran *et al.*, 2013). We found that all differentially expressed ethylene regulatory genes (28) were up-regulated in MEL and FEL tissues, when compared with EEL samples. Up-regulated genes include those having an important role in ethylene biosynthesis (*AMINOCYCLOPROPANE CARBOXYLIC ACID SYNTHASE*: *ACS2*, *ACS6*, A*CS8*, and *ACS11*; Wang *et al.*, 2002) and signalling (*ETHYLENE RESPONSE FACTOR* genes).

Thus, the increase in gene expression of DEGs involved in the biosynthesis and signalling of the stress-related hormones SA, JA, ABA, and ethylene suggests that Arabidopsis MELs and FELs are experiencing higher levels of stress than EELs.

### Expression of stress-related genes in delayed and early ageing mutants

Transcriptomic results showed an enrichment of DEGs involved in stress responses during leaf development. If the increased expression of stress-related genes over time signifies a measure of ageing, we expected that the expression of stress-related genes will be higher in the WT than in delayed ageing mutants, and lower in early ageing mutants. Therefore, we measured the expression of the stress-related genes using qRT-PCR in mutants that display early and delayed senescence, respectively. The *onset of leaf death5* (*old5*) mutation decreases quinolinate synthase activity resulting in higher respiration. Mutants develop normally during early life, but display early ageing (Schippers *et al.*, 2008). The delayed senescence mutant *oresara9* (*ore9*) shows increased longevity during age-dependent natural senescence as a result of a mutation in an F-box protein (Woo, 2001; Schippers *et al.*, 2008).

**Fig. 6.**
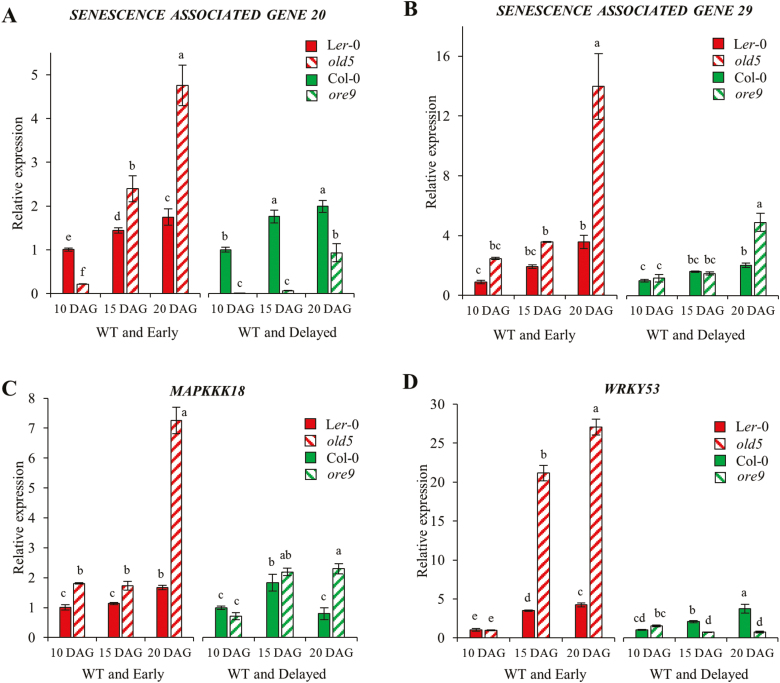
Relative expression of stress-related genes. Expression of genes (A, *SAG20*; B, *SAG29*; C, *MAPKKK18*; D, *WRKY53*) in EEL, MEL, and FEL samples of Arabidopsis ageing mutants. The EEL, MEL, and FEL first rosette leaf pairs were harvested after 10, 15, and 20 DAG, respectively, from Arabidopsis early ageing mutant *old5*, delayed ageing mutant *ore9*, and the WT (L*er*-0 and Col-0). Relative expression was determined by qRT-PCR. The plants were grown under a long-day photoperiod. Solid red and green bars represent relative gene expression in L*er*-0 and Col-0 samples, respectively. Dashed red and green bars represent relative gene expression in *old5* and *old9* mutants, respectively. Gene expression data represent mean values of three biological replicates. Error bars represent the SD, and different letters on each bar represent significant differences at *P*≤0.05 according to ANOVA post-hoc Tukey’s test.

The expression levels of genes involved in senescence (*SAG20*, *SAG29*) and oxidative stress response (*MAPKKK18*, *WRKY53*) were measured by qRT-PCR. The first rosette EEL, MEL, and FEL pairs were used to compare the relative transcript levels between *old5* and its WT L*er*-0, and *ore9* and its WT Col-0. Figure 6A shows that the expression of *SAG20* in *old5* EELs is lower than that in L*er*-0; however, the expression significantly increased by up to 2.5- and 5-fold more than in L*er*-0 in MELs and FELs. As expected, in L*er*-0 MELs and FELs, the expression of *SAG20* also increased, but only 2-fold. In contrast, the expression of *SAG20* in *ore9* was very low in all the sampled tissues, as compared with Col-0. Expression of *SAG29* in *old5* was higher than in L*er*-0 in all leaf samples (Fig. 6B). There was no significant difference in *SAG29* expression between Col-0 and *ore9* EEL and MEL samples, but the expression was ~2-fold higher in *ore9* FELs, as compared with Col-0 FELs. The expression of oxidative stress-responsive genes *MAPKKK18* and *WRKY53* was significantly higher in *old5* MEL and FEL samples than in corresponding leaf samples from L*er*-0 (Fig. 6C, D). No significant difference was observed in expression of *MAPKKK18* in *ore9* EEL and MEL samples, but in FELs the expression was 2.5-fold higher than in the Col-0 control (Fig. 6C). The relative expression of *WRKY53* was found to be significantly lower in *ore9* MELs and FELs than in corresponding leaves of Col-0 (Fig. 6D). Thus, all four marker genes showed elevated expression of *old5*, while the expression of two out of four genes was lower in *ore9*.

### Changes in stress tolerance-related metabolites in Arabidopsis leaves

Several primary metabolites, including amino acids, sugars, and organic acids, have been reported to accumulate and function during environmental stress (Rai, 2002; Kaplan and Guy, 2004; Rosa *et al.*, 2009; Bitrián *et al.*, 2012; Nakabayashi and Saito, 2015; Drincovich *et al.*, 2016; Batista-Silva *et al.*, 2019). The gene expression analysis provided a strong argument that cellular stress increases during leaf development and we expected the abundance of protective metabolites or stress marker metabolites to reflect this too. Therefore, 60 primary metabolites from L*er*-0 WT EEL, MEL, and FEL samples were profiled using GC-MS (Supplementary Fig. S5; Supplementary Table S6).

**Fig. 7.**
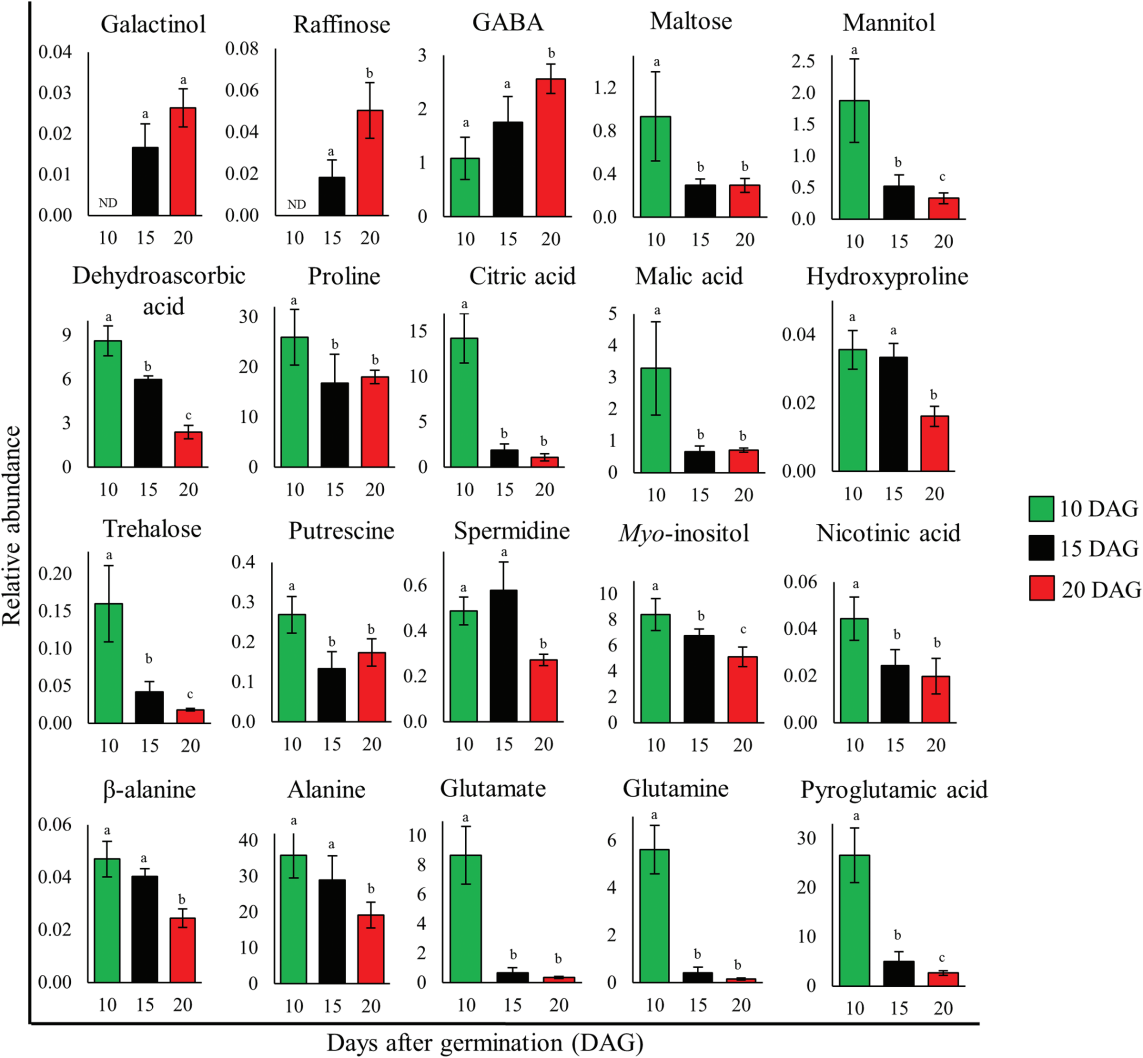
Abundance of stress-responsive primary metabolites in Arabidopsis first rosette leaf samples. Graphs of the indicated metabolites quantified by GC-MS in Arabidopsis WT 10, 15, and 20 DAG first rosette leaves; data are expressed as relative abundance, calculated by normalization of signal intensity to that of ribitol, which was added as an internal standard, and then by fresh weight of the material. Green, black, and red bars represent metabolites in 10, 15, and 20 DAG samples, respectively. Data represent mean values of five biological replicates each. Error bars represent the SD, and different letters on each bar represent significant differences at *P*≤0.05 according to ANOVA post-hoc Tukey’s test; GABA, γ-aminobutyric acid; ND, not detected.

Of the 60 metabolites, 20 are widely reported to accumulate under various stresses and provide protection to plants (Fig. 7; for relevant references see Supplementary Table S7). Of the 40 general metabolites (i.e. those not reported to function in stress adaptation), 24 decreased, while the abundance of three, six, and seven metabolites increased, did not significantly change, or showed different levels, respectively, in MEL and FEL samples compared with EEL samples (Supplementary Fig. S5). Thus, more than half of the general metabolites decreased in abundance, possibly related to a reduction in cellular metabolism as leaves mature. The relative number of stress metabolites that decreased in abundance was significantly larger (*P*<0.05, χ ^2^). Seventeen out of 20 stress metabolites decreased in abundance, namely β-alanine, alanine, glutamic acid, glutamine, hydroxyproline, proline, putrescine, spermidine, pyroglutamic acid, trehalose, *myo*-inositol, mannose, mannitol, citric acid, dehydroascorbic acid, malic acid, and nicotinic acid. Only three metabolites, γ-aminobutyric acid (GABA), raffinose, and galactinol, increased in abundance in MEL and FEL samples (Fig. 7). We next compared the metabolite abundance data with RNA sequencing expression data of genes involved in their biosynthesis (Supplementary Fig. S6; Supplementary Table S8). The data show that the changes in the metabolome can largely be explained by changes in the transcriptome.

### Tolerance to drought stress decreases with age of Arabidopsis leaves

An increased stress gene expression signature and lower abundance of several metabolites that can provide protection against the effects of stress suggest that leaves become less stress tolerant over time. Therefore, Arabidopsis plants of different ages were exposed to drought stress and it was expected that the leaves would become more sensitive to drought stress with age.

**Fig. 8.**
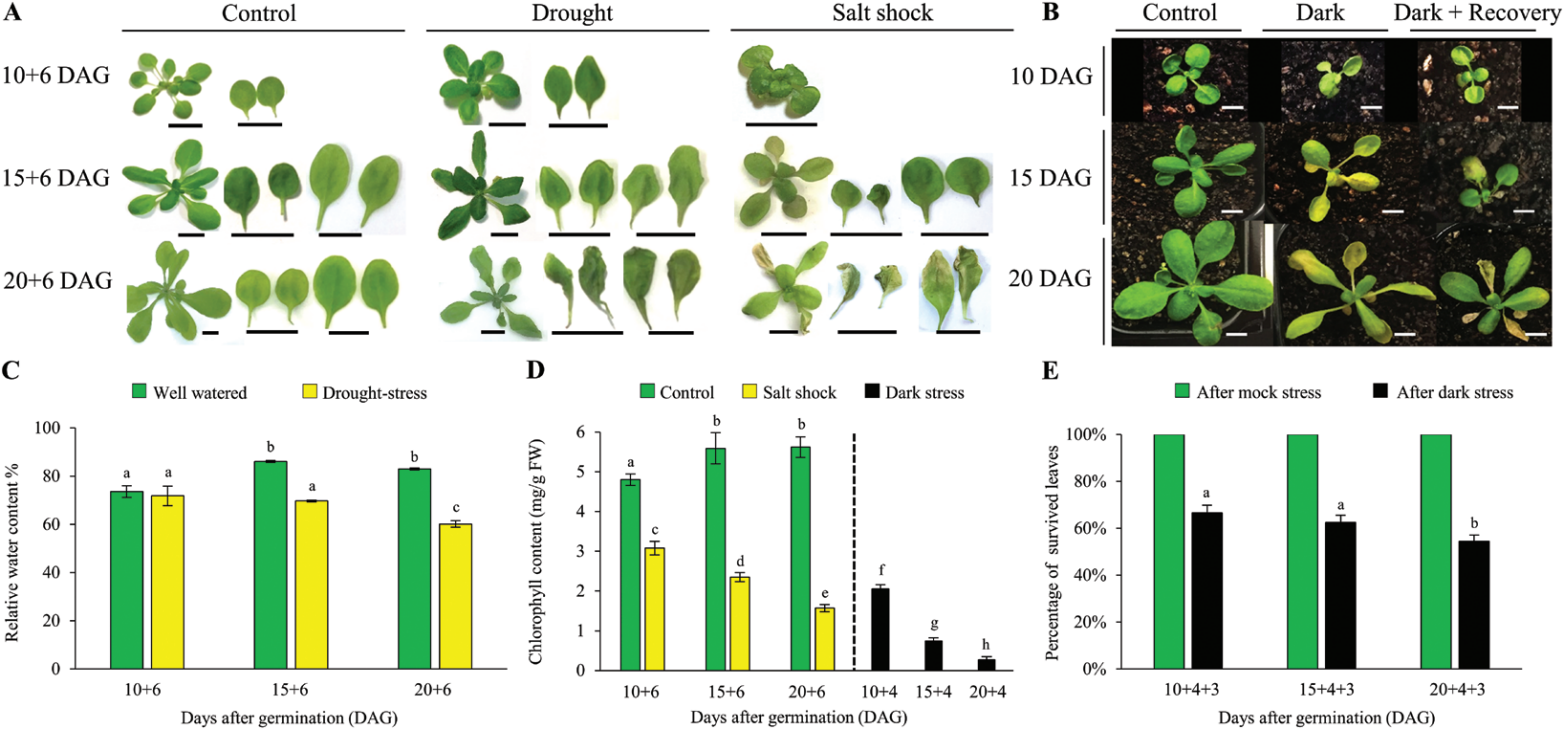
Effect of drought stress, salt shock, and darkness on Arabidopsis WT plants of three different developmental stages. (A) Six days of control, drought, or salt shock treatments were imposed on 10-, 15- and 20-day-old WT plants (10 + 6, 15 + 6, and 20 + 6 DAG) and representative photographs of whole plants and detached first and second rosette leaf pairs are shown. (B) Representative photographs of 10, 15, and 20 DAG plants that were kept in long-day growth conditions (control) or darkness for 4 d (Dark); dark-treated plants were allowed to recover for 3 d in long-day conditions (Dark+Recovery). (C) Relative water content (RWC) of first rosette leaves of control and drought-stressed WT plants after 10 + 6, 15 + 6, and 20 + 6 DAG. Green and yellow bars represent RWC in hydrated plants and drought-stressed plants, respectively. (D) Chlorophyll content was measured in 10 + 6, 15 + 6, and 20 + 6 DAG first rosette leaf pairs of control, salt-shocked, and 4 d dark-treated 10, 15, and 20 DAG WT plants. Green, yellow, and black bars represent chlorophyll content in control, salt-shocked, and dark-treated plants, respectively. (E) Green and black bars represent the percentage of leaves that survived the control (7 d of long-day growth conditions) or dark treatment (4 d of darkness followed by 3 d of long-day conditions), respectively. Leaf survival of dark-treated plants is significantly different (*P*≤0.001, Student’s *t*-test) from control plants, using leaf number data. Data shown in (C–E) represent the mean values of six biological replicates ±SD. Different letters on each bar represent significant differences at *P*≤0.05 determined by ANOVA. Scale bar=10 mm. DAG, days after germination.

Arabidopsis L*er*-0 WT plants were exposed to water deficit conditions, and the effects on the first rosette EELs, MELs, and FELs were examined. The drought-treated plants were well watered every alternate day with a final watering occurring at 8, 13, and 18 DAG, resulting in an expected water deficit from 10, 15, and 20 DAG, respectively (Supplementary Fig. S1A). The plants were subsequently grown for an additional 6 d. Control plants received water every second day for the whole period. After 6 d of drought or control treatment (10 + 6, 15 + 6, and 20 + 6 DAG), the plants were photographed and the leaf RWC was measured. The measured soil field capacity of well-watered pots was ~98%, whereas that of the droughted pots was ~20% (Supplementary Fig. S1B). Figure 8C shows that there is no significant difference in RWC between 10 + 6 DAG leaves from Arabidopsis plants grown in well-watered and drought-stressed conditions. No sign of wilting was observed in the EELs although leaf size appeared reduced compared with the control (Fig. 8A). Symptoms of water stress were clearly visible in 15 + 6 DAG stressed samples, and an ~17% reduction in RWC compared with the control was found. This coincided with a pronounced wilting in MELs of the first rosette leaf pair. The 20 + 6 DAG drought-stressed samples displayed 22% decline in RWC compared with the well-watered plants. This result reflects the degree of wilting, along with the initiation of senescence seen in FELs, and induced leaf wilting in the second rosette leaf pair (Fig. 8A). Although plants of all three developmental stages survived, close inspection of EEL, MEL, and FEL samples, and observed difference in RWC supports the hypothesis that leaves at different ages show distinct adaptive responses to drought stress, where the younger leaves remained green and the older leaves senesced. Therefore, these results suggest that EELs exhibit stronger drought resistance than MEL and FEL first rosette samples.

### Tolerance to salinity shock decreases with age in Arabidopsis leaves

To examine whether tolerance to a different stress is also leaf age dependent, 10, 15, and 20 DAG L*er*-0 plant samples were exposed to a salinity shock. It was expected that with increased age, Arabidopsis leaves would show decreased tolerance to salinity shock, reminiscent of the results from drought stress.

Plants were exposed to salinity shock for 6 d by watering with 300 mM NaCl solution (Supplementary Fig. S2). After 6 d of salinity shock, the stressed and control plants’ rosettes were detached and photographed. The chlorophyll contents in the first rosette EELs, MELs, and FELs was quantified to measure the progression of senescence in control and salinity-shocked samples (Fig. 8D). The overall detrimental effect of salinity shock on plants in all three developmental stages was observed as a reduced leaf number, as well as a decreased rosette leaf area compared with the well-watered plants (Fig. 8A). Salinity stress-induced senescence was not readily visible in the first rosette EEL samples, but chlorophyll quantification showed a substantial reduction in chlorophyll contents (2.06 mg g^–1^ FW) after salinity shock compared with control plants (Fig. 8D; 4.80 mg g^–1^ FW). Chlorophyll contents greatly declined (0.74 mg g^–1^ FW) in salinity-shocked 15 + 6 DAG leaf samples, and this result corresponds to the leaf yellowing observed in the first rosette MEL samples (Fig. 8A). In 20 + 6 DAG salinity-shocked leaves, the chlorophyll level was even lower (0.27 mg g^–1^ FW) and the first rosette FEL appeared dead, while the second rosette leaves appeared yellow (Fig. 8A). These results demonstrate that the EEL samples were less susceptible to salinity shock than MEL and FEL samples, and this is consistent with the previous conclusion that stress tolerance decreases as leaves age.

### Tolerance to dark stress and ability to recover decreases with age in Arabidopsis leaves

Dark treatment imposes a nutrient stress and if stress tolerance generally decreases with leaf age, then the sensitivity of leaves to dark stress should also increase with age. To determine the effect of dark stress and subsequent recovery in light of differently aged leaves, L*er*-0 plants at 10, 15, and 20 DAG were exposed to darkness for 4 d and then returned to a long-day photoperiod for 3 d (Fig. 8B). To understand the progression of senescence after 4 d of darkness, the total chlorophyll level was measured from the first rosette EEL, MEL, and FEL samples. The ability of plants to recover after stress was measured by calculating the percentage of leaves that survived after dark recovery, over those present before the dark treatment. Chlorophyll content declined in all leaf samples that were exposed to darkness for 4 d (10 + 4, 15 + 4, and 20 + 4 DAG). However, chlorophyll content progressively and significantly decreased in leaves of increasing age (Fig. 8D). Survival after 3 d of recovery in 10 + 4+3 DAG plant samples was the highest, with 66% of leaves surviving, while in 15 + 4+3 DAG plant samples, 62% of MELs survived and only 54% of FELs recovered in 20 + 4+3 DAG plant samples (Fig. 8E). Taken together, the percentage of leaf survival decreased with age after dark stress treatment. This study concludes that the tolerance to dark stress decreases with leaf age, as does the ability to recover, which was found to be the highest in EELs, followed by MEL and FEL samples.

## Discussion

### Senescence-inducing ARCs control the induction of leaf senescence in response to stress

Leaf senescence is a destructive process, but it releases valuable nutrients that can benefit plant growth in the next season or generation (Thomas, 2013). Leaf senescence is also induced by stress (Kanojia and Dijkwel, 2018), suggesting that the senescence process can provide nutrients to allow plant survival, or decrease the plant’s total demand for nutrients or water. After induction of senescence, the senescence process itself also modulates the oxidative stress level in plants (Breeze *et al.*, 2011; Rogers and Munné-Bosch, 2016). Nevertheless, stress-induced leaf senescence is tightly regulated and strictly limited to the older leaves, where there appears to be a strong positive correlation between the age of an individual leaf on a plant and the chance that this particular leaf senesces in response to the stress (Fig. 8). While to the best of our knowledge this has not been quantified, examples of such age-related induction of stress-induced senescence can be found in the literature (e.g. note figures in Wang *et al.*, 2012; Moustaka *et al.*, 2015; Zhao *et al.*, 2016; Kanojia and Dijkwel, 2018). Thus, the ability of individual leaves to respond to stress with senescence appears to be controlled by ARCs, which occur in still developing leaves, well before developmental senescence is induced. Here, we refer to these ARCs as senescence-inducing ARCs and, to begin to understand those, we first analysed transcriptomic changes that take place in young, expanding leaves.

Among genes that were up-regulated during early leaf development, stress-related genes were most enriched (764/805; Fig. 3) and studies have found that the onset of senescence also coincides with the activation of stress-related genes (Breeze *et al.*, 2011; Guo and Gan, 2012; Kong *et al.*, 2013; Jehanzeb *et al.*, 2017). When we compared all up-regulated genes with those of Breeze *et al.* (2011) (clusters 27–48), we found a significant overlap (*P*<0.05 as determined by Fisher’s exact test) and both gene sets contained a high number of GO terms enriched in terms related to stress (Supplementary Table S9). Nevertheless, the relative number of genes with GO terms related to senescence (‘leaf-senescence’, ‘organ senescence’, and ‘ageing’) was higher in the data of Breeze *et al.* (2011) (*P*<0.05, χ ^2^), consistent with their different phase in leaf development. Thus, our data show that the activation of stress-related genes occurs already in expanding leaves, well before the onset of senescence, and is consistent with the idea that a continuous increase in stress levels is part of the normal leaf developmental programme.

### Oxidative stress-related genes are up-regulated during Arabidopsis leaf development

Most up-regulated stress-associated genes were linked to the GO term ‘responsive to oxidative stress’ (40%) and ‘other stress responses’ (55%) (Fig. 4B; Supplementary Table S5). Up-regulated genes include NAC and WRKY transcription factor genes, such as *WRKY57*, *WRKY33*, *WRKY27*, *WRKY25*, *NAC019*, and *NAC13*, that are up-regulated by stress and are well documented to be regulated by reactive oxygen species (ROS) levels (Balazadeh *et al.*, 2008; Gujjar *et al.*, 2014; Hoang *et al.*, 2017); two genes encoding cysteine-rich RLK receptor-like protein kinases, *CRK6* and *CRK7*, which mediate extracellular signalling in response to ROS production and expression of which is elevated when exposed to oxidative stress (Idänheimo *et al.*, 2014); a calmodulin-binding-like cytoplasmic kinase 1 (*CRCK1*) gene that is involved in transduction of stress signals in plants (Yang *et al.*, 2004); and the zinc finger protein ZAT12-encoding gene, expression of which increases in response to biotic and abiotic stress and which plays a crucial role in regulation of ROS and stress signalling (Davletova *et al.*, 2005). Up-regulation of these genes shows that cellular ROS levels increase during leaf expansion, possibly as a result of the maturation of highly active metabolic pathways. This coincided with a cellular response to oxidative stress as evidenced by the up-regulation of antioxidant-related genes (Fig. 3B). Also coinciding with the increase in expression of stress-associated genes, expression of genes related to biosynthesis and signalling of the stress hormones JA, ABA, SA, and ethylene increased (Fig. 5). Thus, the RNA sequencing data explain well that leaf maturation is inherently coupled to increased oxidative stress.

RNA sequencing data furthermore revealed the up-regulated expression of several genes implicated in the regulation of senescence during leaf expansion. These include the SAG genes *SAG2*, *SAG20*, *SAG21*, *SAG29*, *SAG113*, and *SAG13* (Gepstein *et al.* 2003), genes involved in the regulation of senescence, *ORE1*, *WRKY53*, and genes related to mitogen-activated signalling pathways (*MPK3*, *MPK11*, *MKK1*, and *MAPKKK18*). Nevertheless, these genes have also been reported to regulate stress. For example, *ORE1* encodes a well-characterized NAC transcription factor that induces leaf senescence under stress conditions (Woo *et al.*, 2004; Balazadeh *et al.*, 2011; Zhou *et al.*, 2013) and expression of the senescence regulatory gene *WRKY53* is highly up-regulated in response to oxidative stress (Miao *et al.*, 2004; Miao and Zentgraf, 2007; Xie *et al.*, 2014). Moreover, transgenic plants overexpressing the *JUB1* NAC transcription factor gene exhibited extended leaf longevity, strongly delayed reproductive growth, and greater tolerance to stress-inducing treatments (Wu *et al.*, 2012), while Arabidopsis lines overexpressing *AtERF019* showed enhanced drought tolerance and delayed natural senescence compared with WT plants (Scarpeci *et al.*, 2017). Thus, our findings are consistent with the well-described relationship between oxidative stress and the onset of senescence (for reviews, see Juvany *et al.*, 2013; Jajic *et al.*, 2015). Crucially, we found that the early and late senescence mutants *old5* and *ore9* exhibited earlier and delayed up-regulation of the expression of oxidative stress-related genes, respectively (Fig. 6). Therefore, the developmentally controlled increase in oxidative stress levels—well before the visible onset of senescence—may constitute a senescence-inducing signal and we refer to these changes as senescence-inducing ARCs.

### A decrease in stress tolerance-related metabolites explains why leaves gradually become more susceptible to stress

Plants have evolved various protective mechanisms to minimize the adverse effect of environmental stress, and the accumulation of specific primary metabolites is regarded as an adaptive response to stress (Sousa and Sodek, 2002; Fraire-Velazquez and Emmanuel, 2013; Lamalakshmi Devi *et al.*, 2017; Isah, 2019). We found that in developing leaves most metabolites that provide protection against various environmental stresses decrease in abundance (Fig. 7; Supplementary Table S7). Importantly, the metabolomics data provide a possible explanation for why oxidative stress-related genes are up-regulated during Arabidopsis leaf development: The polyamines putrescine and spermidine accumulate in response to a range of abiotic stresses to mitigate cellular damage (Liu *et al.*, 2015; Zhang *et al.*, 2015), and were shown to reduce ROS abundance and delay the senescence process (Woo *et al.*, 2013; Karimi *et al.*, 2017; Chen *et al.*, 2019). The amino acids alanine, β-alanine, proline, and glutamate decreased during leaf development and these amino acids and some derivatives thereof have been shown to provide protection against various environmental stresses (Sharma and Dietz, 2006; Martinelli *et al.*, 2007; Hayat *et al.*, 2012; Majumdar *et al.*, 2016; Batista-Silva *et al.*, 2019; Parthasarathy *et al.*, 2019).

Nicotinic acid provides stress resistance in plants by protecting the cells against oxidative damage (Hussein *et al.*, 2014; Berglund *et al*., 2016, 2017). Dehydroascorbic acid plays an important role in plant adaptation to environmental stresses (Akram *et al.*, 2017; Chang *et al.*, 2017; Hasanuzzaman *et al.*, 2019) and, by means of the dehydroascorbate reductase (DHAR) reaction (Smirnoff, 1996; Smirnoff and Wheeler, 2000), can be reduced to ascorbic acid, one of the most important antioxidants in plants (Conklin and Barth, 2004; Akram *et al.*, 2017). Citric acid and malic acid are products of the tricarboxylic acid cycle and are known to improve the activity of antioxidant enzymes under stressed conditions (Zhou *et al.*, 2012; Hu *et al.*, 2016; Abdellatif and Ibrahim, 2018; Sun *et al.*, 2019).

Several sugars have been reported to provide tolerance to harsh environments (Rosa *et al.*, 2009; Boriboonkaset *et al.*, 2012; Gechev *et al.*, 2013; Wei *et al.*, 2019) and are linked to stress-induced ROS accumulation, where sugars act as ROS scavengers to protect cells against oxidative damage (Bolouri-Moghaddam *et al.*, 2010; Keunen *et al.*, 2013; Van Den Ende and Peshev, 2013). Our data are consistent with those of others who found that sugars such as trehalose, maltose, *myo*-inositol, and mannitol play roles as protectants against oxidative stress and accumulated to lower levels in more developed leaves (Fig. 7; Stoop *et al.*, 1996; Kaplan and Guy, 2004; Bae *et al.*, 2005; Cortina and Culiáñez-Macià, 2005; Iordachescu and Imai, 2008; Valluru and Van den Ende, 2011; Kaya *et al.*, 2013; He *et al.*, 2017; Khurana *et al.*, 2017).

We found that three stress-related metabolites, namely raffinose, galactinol, and GABA, increased during development. These metabolites are known to accumulate during stress and provide protection against oxidative stress by preventing ROS accumulation (Nishizawa-Yokoi *et al*., 2008*a*, *b*; Song *et al.*, 2010; Elsayed *et al.*, 2014; Lahuta *et al.*, 2014; Pluskota *et al.*, 2015; Li *et al.*, 2016; Yong *et al.*, 2017). Although we do not know the precise roles of these metabolites during leaf development, their accumulation may balance the decrease of others to allow maintenance of redox homeostasis.

Together, the results suggest that the lower level of the majority of stress tolerance-related metabolites in older leaves may provide reduced protection against ROS-related stress and this may induce the oxidative stress gene expression signature in older leaves.

### Integration of leaf age and stress determines the stress response

The effect of stress on individual leaves strictly depended on the age of those leaves. Young leaves were remarkably resistant to various stresses, displaying little sign of senescence, while old leaves readily died (Fig. 8). Our results are well supported by the stress responses observed in previous studies: young detached *Xanthium* leaves did not wilt after drought stress, but mature leaves showed signs of leaf dryness and stress-induced ABA accumulation (Cornish and Zeevaart, 1984). Moreover, young cucumber leaves displayed remarkable resistance to sulfur dioxide stress, whereas mature leaves were more susceptible (Sekiya *et al.*, 1982). More recently, Wang *et al.* (2012) showed that young rice leaves adapt well to alkaline stress, while this treatment damaged the cell membranes of adult rice leaves and caused a reduction in leaf chlorophyll pigment. Paraquat sprayed on young Arabidopsis leaves resulted in reduced ROS accumulation due to higher antioxidant activity, while adult leaves exhibited damage from high ROS abundance and reduced scavenging activity (Moustaka *et al.*, 2015).

While our work does not demonstrate a causal relationship between developmental changes in oxidative stress tolerance and leaf senescence, our results help to hypothesize why stress responses are age dependent. We propose that young leaves have experienced few ARCs and hence are subject to low intrinsic oxidative stress levels, while ARCs in fully expanded leaves result in increased oxidative stress levels. Externally imposed stress then adds even more oxidative stress (Apel and Hirt, 2004; Choudhury *et al.*, 2017; Xie *et al.*, 2019), and the outcome for the leaf, namely life (resistance) or death (senescence), depends on the combined internally and externally induced oxidative stress levels. The stress tolerance of individual leaves, therefore, strictly depends on the age of that leaf. Induced senescence in older leaves can then support the survival of the younger leaves by allowing recycling of nutrients from old to young leaves and reduction of total plant nutrient requirements. Certainly, while leaf senescence is a destructive process, efficient senescence increases viability of the whole plant and survival to the next season or generation (Lim *et al.*, 2007; Guiboileau *et al.*, 2010). That senescence of older leaves coincides with increased stress tolerance of the whole plant has been clearly demonstrated by Zhao *et al.* (2016): Arabidopsis lines overexpressing the ABA receptor gene *PYL9* displayed drought tolerance at the expense of early senescence of the older leaves. Therefore, stress tolerance in young leaves and stress susceptibility in old leaves both appear to contribute to survival of the whole plant.

**Fig. 9.**
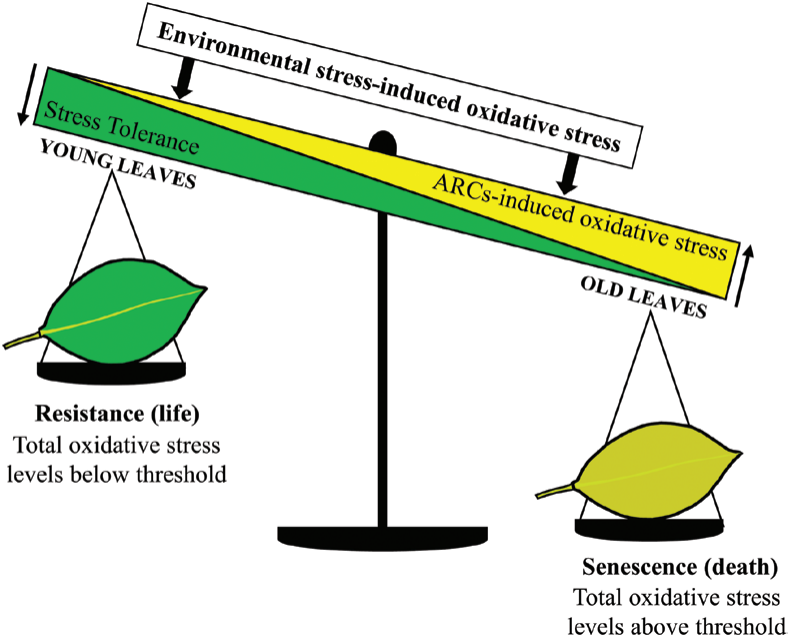
A tentative model explaining how ARCs allow efficient integration of individual leaf age into the stress response. Leaf survival or death under stress is determined by the age of a leaf. In young leaves, only a few senescence-inducing ARCs have occurred, resulting in low intrinsic oxidative stress levels. During leaf development, occurrence of senescence-inducing ARCs increases the intrinsic oxidative stress level. Oxidative stress incurred by environmental stress adds to the total stress levels. If total oxidative stress levels are below a threshold level, the leaf will induce a resistance response and survive. As a result of ARCs, old leaves will already have high intrinsic stress levels and additional environmental stress is more likely to cause the total stress levels to go beyond the threshold, resulting in senescence of the leaf. ARCs, age-related changes.

Thus, a leaf’s reaction to stress is primarily determined by the age of the leaf. Leaf age—through the action of senescence-inducing ARCs—is a checkpoint that determines leaf survival or death in response to stress. The model in Fig. 9 illustrates how the age of a leaf is integrated into the stress response and explains why many early (i.e. *cpr5/old1*, *old5*, *jub1*, and *argos*) and delayed (i.e. *aaf*, *saur36*, *ore1*, and *ore9*) ageing mutants display increased and decreased oxidative stress levels, respectively (Hu *et al.*, 2003; Jing *et al.*, 2008; Schippers *et al.*, 2008; Sharabi-Schwager *et al.*, 2009; Chen *et al.*, 2012; Wu *et al.*, 2012; Hou *et al.*, 2013). The model also predicts that the continuous ARC-induced increase in intrinsic stress levels ensures a timely and certain death of individual leaves, even in the absence of external stress. Such a tight correlation between leaf development and senescence may render it impossible to block leaf senescence without also disrupting leaf development.

## Supplementary data

The following Supplementary data are available at *JXB* online.

Table S1. RNA sequencing QC statistics.

Table S2. Primer sequences of gene markers used for expression analysis.

Table S3. List of differentially expressed genes.

Table S4. Enriched GO terms.

Table S5. List of differentially expressed genes responsive to stress, oxidative stress, and senescence.

Table S6. Raw data of all metabolites identified by GC-MS analysis.

Table S7. List of metabolites reported to function in stress tolerance.

Table S8. List of primary metabolites and related genes from RNA sequencing data.

Table S9. List of genes with GO terms related to ‘leaf-senescence’, ‘organ senescence’, and ‘ageing’ compared with Breeze *et al.* (2011).

Figure S1. Watering schedule during drought stress and measured soil field capacity in well-watered pots and drought-stressed pots.

Figure S2. Watering schedule during salt shock.

Figure S3. Chlorophyll and electrolyte leakage measurement of first rosette leaf pairs.

Figure S4. Validation of RNA sequencing results.

Figure S5. Primary metabolite profiling.

Figure S6. Validation of metabolomic results.

eraa347_suppl_Supplementary_File001

eraa347_suppl_Supplementary_File002

## Data Availability

The RNA sequencing data are available under NCBI Bioproject accession number PRJNA557609. All other data supporting the findings of this study are available within the paper and its supplementary data.

## **Abbreviations**:

ABA: abscisic acid
ARC: age-related change
DAG: days after germination
DEG: differentially expressed gene
EEL: early expanding leaf
FEL: fully expanded leaf
GO: Gene Ontology
JA: jasmonic acid
MEL: mid expanding leaf
RWC: relative water content
SA: salicylic acid
