# Supplementary material for: Developmentally controlled changes during Arabidopsis leaf development indicate causes for loss of stress tolerance with age
Source: J Exp Bot. 2020 Aug 28;71(20):6340–54. doi: 10.1093/jxb/eraa347 (PMC7586751; doi:10.1093/jxb/eraa347)
Supplement: eraa347_suppl_Supplementary_File001 [file eraa347_suppl_supplementary_file001.pdf]

Table S1. RNA sequencing QC statistics. Read QC stats generated using an in-house script, after filtering for rRNA and pseudo alignment stats using kallisto v0.42.4.

Sample description	Reads	% reads having >= 75% bases >= Phred 20	rRNA filtered reads	% rRNA reads	Pseudo aligned reads	% Pseudo aligned reads
L*er*-0 10 DAG rep. 1	25,225,200	99.11	25,048,498	0.70	23,912,520	95.46
L*er*-0 10 DAG rep. 2	32,974,327	99.31	32,706,109	0.81	31,124,610	95.16
L*er*-0 10 DAG rep. 3	27,576,878	99.28	27,267,880	1.12	26,095,866	95.70
L*er*-0 15 DAG rep. 1	31,313,194	99.33	30,952,487	1.15	29,780,083	96.21
L*er*-0 15 DAG rep. 2	26,718,331	99.25	26,503,135	0.81	25,512,406	96.26
L*er*-0 15 DAG rep. 3	26,326,789	99.16	25,845,646	1.83	24,871,182	96.23
L*er*-0 20 DAG rep. 1	27,731,359	99.38	27,466,145	0.96	26,417,210	96.18
L*er*-0 20 DAG rep. 2	29,978,924	99.49	29,814,566	0.55	28,644,308	96.07
L*er*-0 20 DAG rep. 3	29,020,922	99.48	28,737,149	0.98	27,614,477	96.09

DAG = Days after germination; Rep. = Replicate.

Table S2. Primer sequences of gene markers used for expression analysis.

Gene name	AGI	Forward (5'-3')	Reverse (5'-3')
RBOHD	AT5G47910	CCACTCGTGTGGGACGATATTC	ACGACACCAAGTGGTTCAGAAATG
WRKY53	AT4G23810	CTGTTGCTGAGACTAACGAGAT	CCTCCATCGGCAAACTCTT
SAG13	AT2G29350	CTTGTCACTGGTGGCTCTAA	CAGTTTCCATGAGTTTCACTCG
FSD3	AT5G23310	CCACTCGTGTGGGACGATATTC	ACGACACCAAGTGGTTCAGAAATG
BARD1	AT1G04020	GTTAAAGCACAGAACCATCAG	CATTGCATCAGAAGCGGTGATGTC
MRE11	AT5G54260	AACAAATCTCAGCCTCGGGTT	AGAAGTTGTTCCGCTTGAGAGGTC
SAG20	AT3G10985	GGTAACGTTGTTGCTGGACGAC	TTCCCACACGGAGGAACAATGC
SAG29	AT5G13170	TAAGCGCCGTTATGTGGTTCGC	ATCCCACCACGTTTGGAATCGC
MAPKKK18	AT1G05100	AAGCAACGTGTTGGTCGGAGAG	CGGTTCAACCCATTTCGCACAC
UPL7	AT3G53090	TTCAAATACTTGCAGCCAACCTT	CCCAAAGAGAGGTATCACAAGAGACT
ACTIN2	AT3G18780	TCCCTCAGCACATTCCAGCACAT	AACGATTCCTGGACCTGCCTCATC
TUB2	AT5G62690	GCCAATCCGGTGCTGGTAACA	CATACCAGATCCAGTTCCTCCTCCC

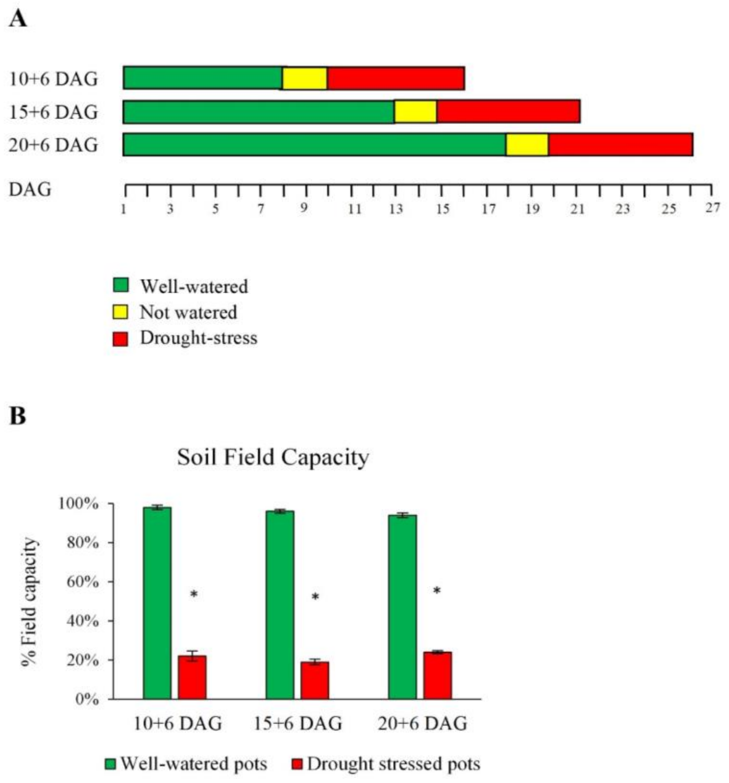

Figure S1. Watering schedule during drought stress. **A.** After germination, WT plants were watered equally every alternate day and watering was stopped after 8, 13 and 18 DAG to initiate the drought stress after 10, 15 and 20 DAG, respectively. Physiological measurements and pictures were taken after 6 days of drought stress in each time point. Watering was not stopped for the control plants. **B.** Measured soil field capacity (SFC) in well-watered pots and drought-stressed pots after 10+6, 15+6 and 20+6 DAG plants. Green bars represent SFC in hydrated plants and yellow bars represent SFC in drought-stressed plants. Results are represented as means and standard deviations from 6 pots. Asterisks (*) indicate values that are significantly different at $P \leq 0.05$ using Student's *t*-test, between the hydrated and drought-stressed plants.

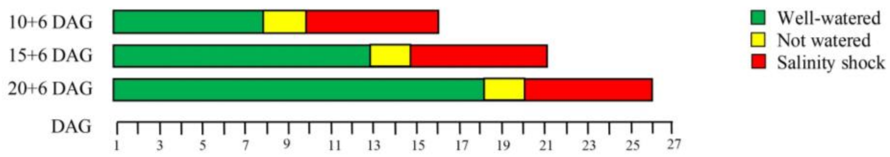

Figure S2. Watering schedule during salt shock. *Arabidopsis* plants were normally watered till 8, 13 and 18 DAG and then exposed to salt shock for 6 days by watering with 300 mM of NaCl solution in equal volume at 10, 15 and 20 DAG. Control plants were watered with water.

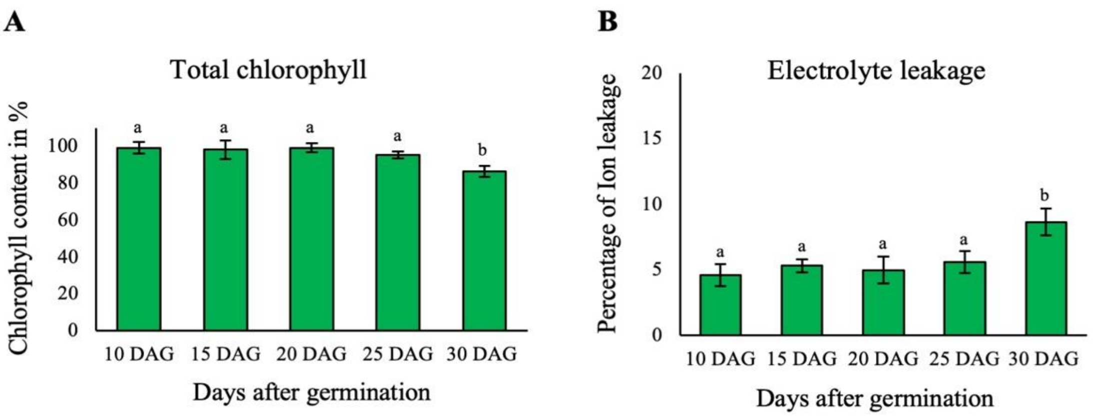

Figure S3. Chlorophyll and electrolyte leakage measurement of first rosette leaf pairs. L*er*-0 WT plants were grown under long-day growth conditions and chlorophyll content (A) and electrolyte leakage (B) was measured in first rosette leaf pair at 10, 15, 20, 25 and 30 DAG. Different letters on each bar represents significant differences at $p \leq 0.01$ determined by ANOVA.

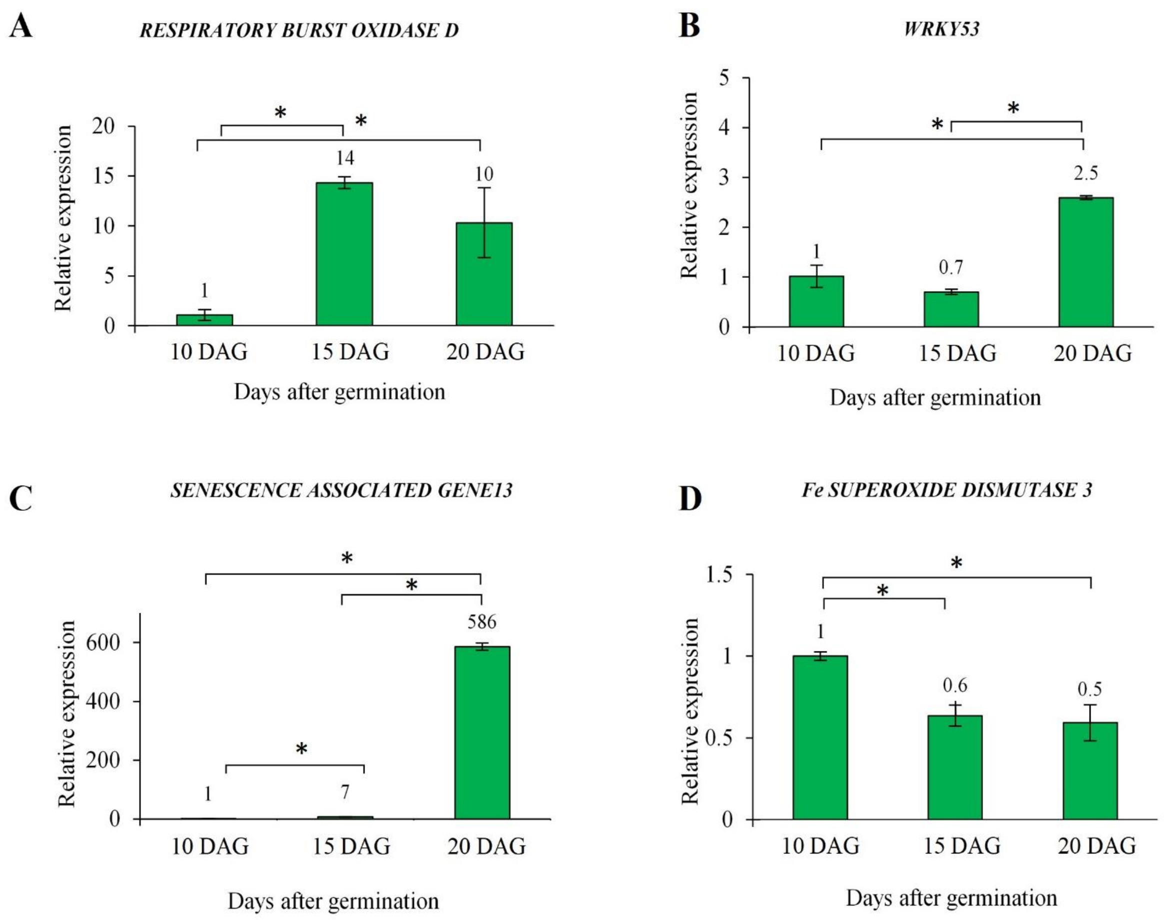

Figure S4. Validation of RNA sequencing results. Transcript expression pattern of selected genes (*RBOHD, WRKY53, SAG13* and *FSD3*) from RNA sequencing data was verified by qRT-PCR in *Arabidopsis* WT 10, 15 and 20 DAG first rosette leaf samples. The plants were grown under long-day photoperiod (16-hour light: 8-hour dark). Gene expression data represent mean values of three biological replicates. Asterisks (*) indicate values that are significantly different at $P \leq 0.05$ using a Student's *t*-test between the indicated samples.

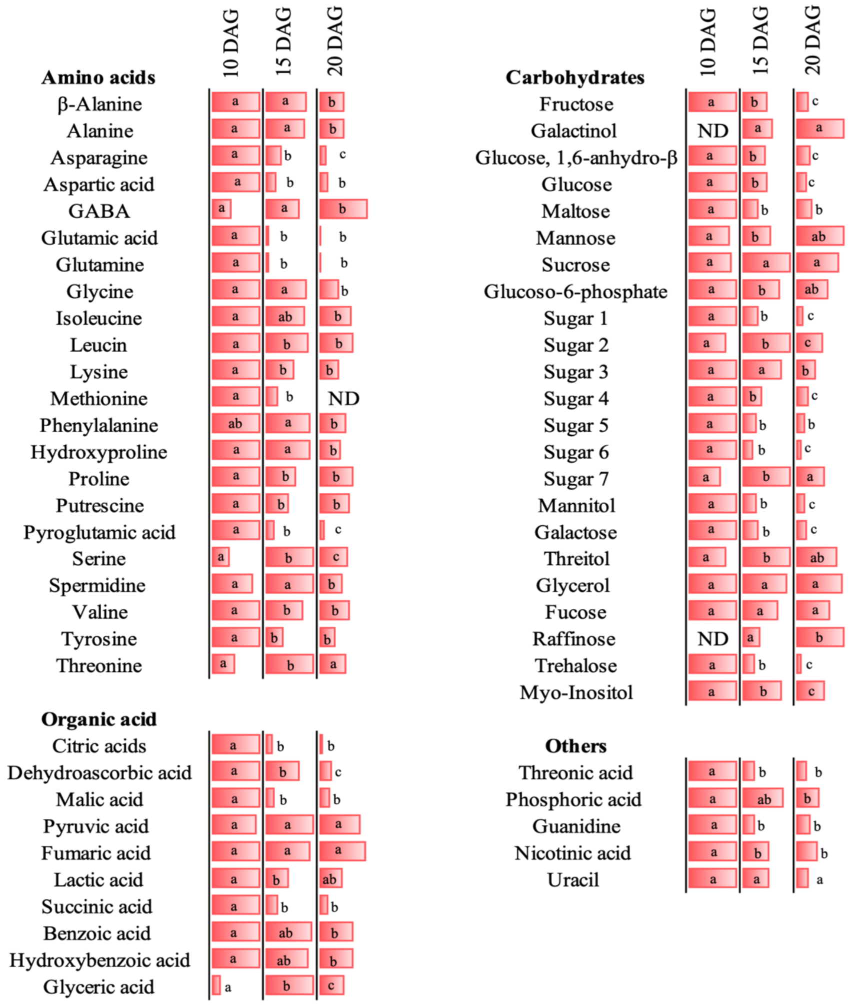

Figure S5. Primary metabolite profiling. Data bars of primary metabolites in *Arabidopsis* L*er*-0 WT 10 DAG, 15 DAG and 20 DAG first rosette leaf samples. The primary metabolites include amino acids, carbohydrates, organic acids and other compounds. Bars reflect the relative abundance in 10 DAG, 15 DAG and 20 DAG samples, calculated by normalization of signal intensity to that of ribitol, which was added as an internal standard, and then by fresh weight of the material. Different letters on each bar represent significant differences at $P \leq 0.05$ according to ANOVA-Post Hoc Tukey's test; GABA, γ-aminobutyric acid; ND, not detected; sugar 1 – sugar 7 are unidentified sugars.

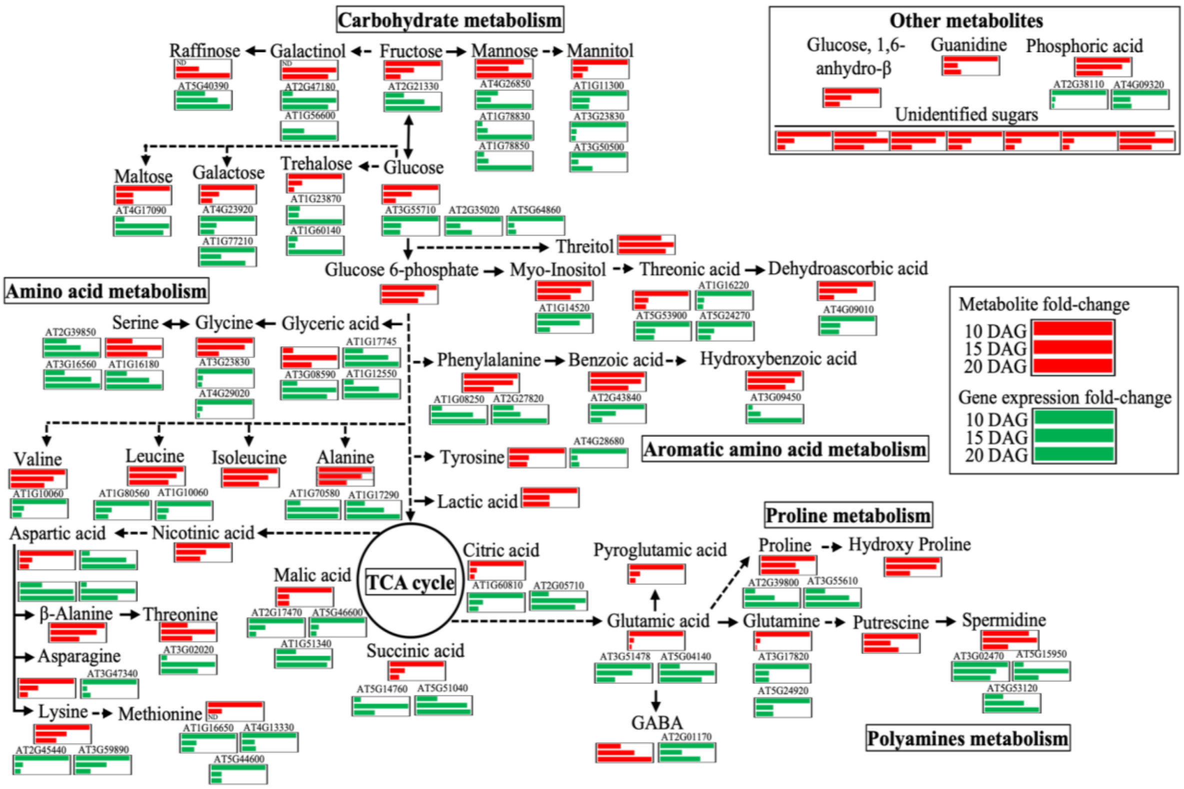

Figure S6. Validation of metabolomic results. Comparison of significantly different primary metabolites and related genes from RNA-sequencing data. Data bars of indicated metabolites and genes are expressed as fold-change in *Arabidopsis* L*er*-0 WT 10 DAG, 15 DAG and 20 DAG first rosette leaf samples. The primary metabolic pathways include carbohydrate metabolism, amino acid metabolism, aromatic amino acid metabolism, proline metabolism, TCA cycle and polyamines metabolism. Red and green bars reflect the fold change: red indicates metabolite, and green indicates gene. GABA, γ-aminobutyric acid; ND, not detected.
